# Calcinosis circumscripta of the breasts: The deeper meaning

**DOI:** 10.4102/sajr.v27i1.2730

**Published:** 2023-09-27

**Authors:** Tanusha Sewchuran, Joel M. Kabeya

**Affiliations:** 1 Department of Radiology, Faculty of Health, Greys Hospital, University of KwaZulu-Natal, Pietermaritzburg, South Africa; 2 Department of Radiology, Greys Hospital, Pietermaritzburg, South Africa

**Keywords:** calcinosis circumscripta, breast calcinosis, benign breasts calcification, mammography, HRCT

## Abstract

Calcinosis circumscripta involving the breasts usually hints at an underlying systemic cause, most commonly connective tissue disorders such as scleroderma or dermatomyositis. Localised scleroderma, also known as morphoea, is not usually associated with the systemic anomalies affecting the lungs and gastrointestinal system, but does manifest as calcified dermal and subdermal sclerotic plaques. Histological confirmation with skin biopsy is diagnostic.

**Contribution:**

Recognition of the atypical, bizarre, linear dystrophic calcifications on mammography should direct appropriate systemic investigations.

## Introduction

Calcinosis refers to the deposition of calcium in the skin and subcutaneous tissues and can either be localised in the form of calcinosis circumscripta or diffuse, calcinosis universalis.

## Patient presentation

**FIGURE 1:**
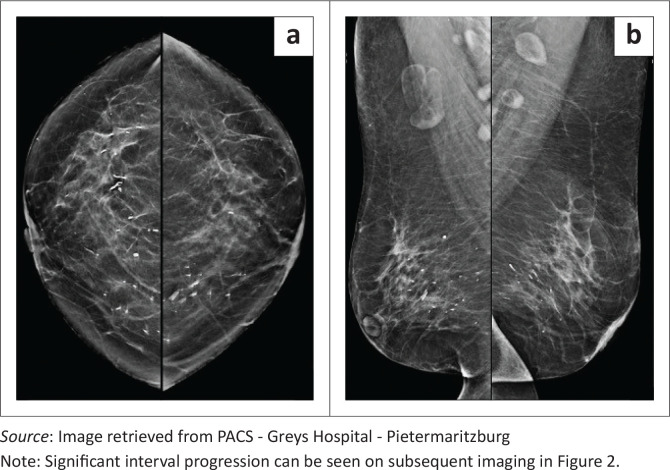
Baseline mammography in 2017 demonstrates the benign-appearing calcifications bilaterally on the (a) CC and (b) MLO views.

A 61-year-old female presented for mammography with a history of *‘peau d’orange’* and multiple ‘lumps’ translated as multiple suspected intraductal plaques palpable on clinical review. Baseline mammography in 2017 was evaluated as BIRADS 2, with several secretory type calcifications observed bilaterally (Figure 1). On further interrogation, the patient was known to dermatology with an undifferentiated connective tissue disorder, Raynaud syndrome and an anti-neutrophil cytoplasmic antibody (ANCA)-negative panniculitis or vasculitis. The diagnosis was confirmed with skin punch biopsy of an upper chest lesion, histologically proven as an old non-specific panniculitis, healing with fibrosis and focal calcification. No evidence of lupus was found. Biochemically, the patient’s calcium and phosphate levels were within the normal range.

**FIGURE 2:**
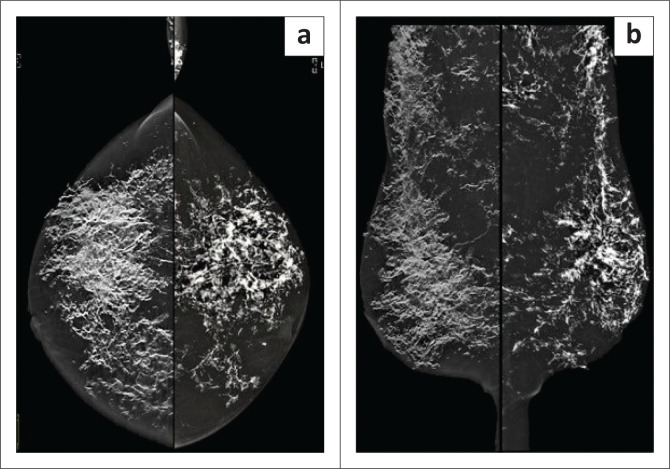
The 2021 (a) craniocaudal (CC) and (b) mediolateral oblique (MLO) views eloquently demonstrate the striking bizarre linear dystrophic type calcifications in both breasts.

**FIGURE 3:**
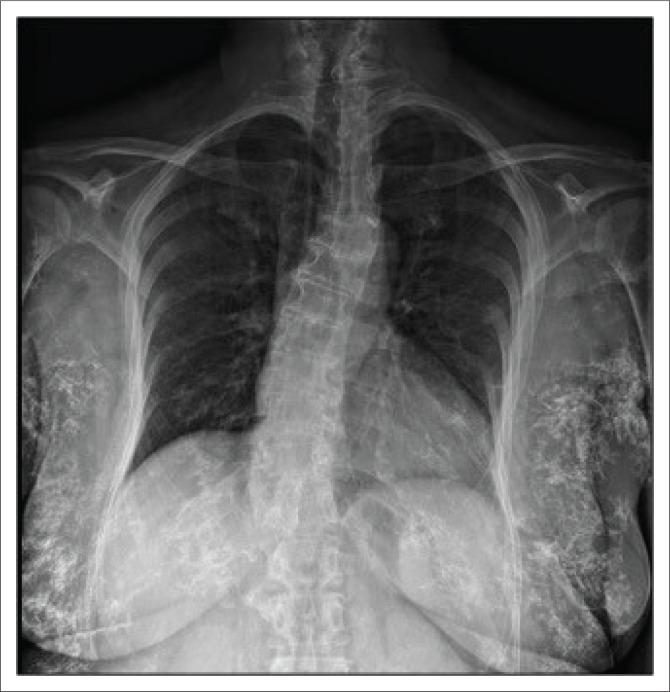
The chest radiograph (CHR) reveals the extensive bizarre breast calcifications with contrasting insignificant pulmonary changes.

Imaging of the chest and breasts was performed subsequently. Mammography demonstrated marked interval progression in the previously observed benign calcifications, now seen to extensively involve the stromal tissue of both breasts and the subcutis (Figure 2). The calcifications appeared bizarre with a coarse linear morphology. The pectoralis muscles were relatively spared. No overt primary or secondary features of malignancy were identified. On further evaluation, scattered foci of mass-like calcifications were found to involve the subcutaneous tissues of the chest wall, axillae and abdomen (Figure 3). Ultrasound evaluation of the chest and abdominal wall revealed similar findings to that seen on breast ultrasound.

**FIGURE 4:**
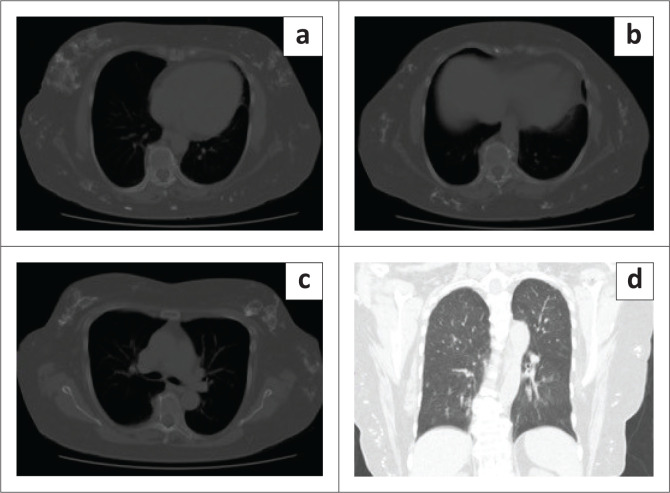
Corresponding axial HRCT on bone window (a, b, c) demonstrate scattered foci of mass-like calcifications in the breasts and the subcutaneous tissues of the chest wall and axillae and coronal (d) demonstrates lung fields without imaging findings suggestive of interstitial lung disease.

**FIGURE 5:**
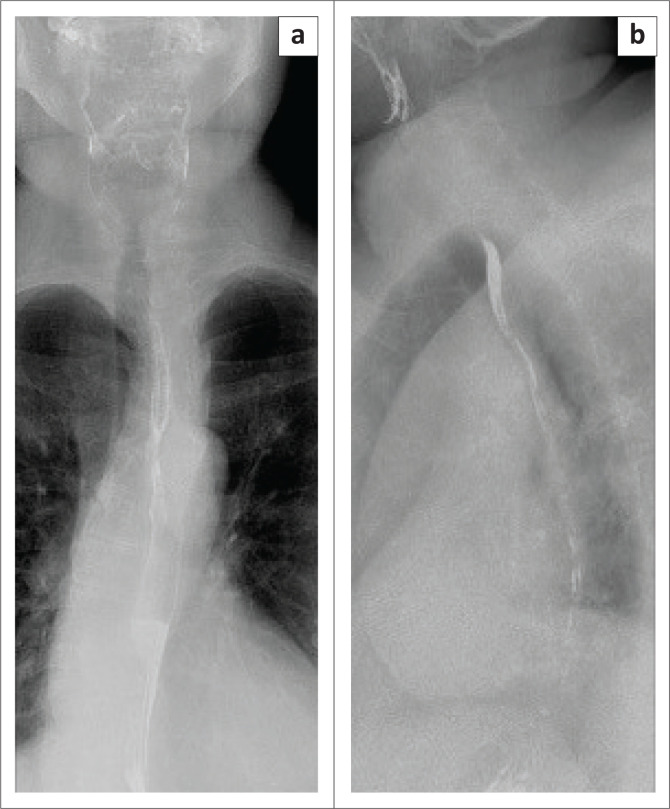
Contrast swallow (a) anteroposterior, demonstrates an unremarkable oesophageal outline without any features of dysmotility. The lateral (b) did not demonstrate any oesophageal pathology.

High resolution CT of the lungs performed thereafter, eloquently demonstrated the breast parenchymal changes with no interstitial lung changes (Figure 4). Similar appearing dystrophic calcifications were seen throughout the subcutis of the chest and abdomen. There were no discernible lung changes as compared with previous HRCT chest imaging from 2017. Contrast swallow was also normal at this stage, making CREST syndrome (calcinosis, Raynaud’s, oesophageal dysmotility, sclerodactyly and telangiectasia) and dermatomyositis less likely differentials (Figure 5).

## Discussion

The deposition of calcium in the skin and subcutaneous tissues, termed calcinosis, is generally attributed to either local or systemic causes.^1,2^ It can be differentiated as calcinosis circumscripta, which refers to more localised deposits in the cutis or subcutis, as compared to calcinosis universalis which includes muscle and tendon deposition.^1,2^ This calcinosis is a rare but documented cutaneous manifestation, first described in animals.^1,3^ It has also been established in patients with dermatomyositis and systemic lupus.^3,4^ Another recognised cause is localised scleroderma (LS) or morphoea, first described in 1878 by Weber.^1^ Localised scleroderma may be distinguished from systemic scleroderma by the lack of associated gastrointestinal, renal, cardiac and pulmonary anomalies.^5^ Localised scleroderma or morphoea can be broadly classified into plaque morphoea, generalised morphoea, linear morphoea or deep morphoea.^6^ Plaque morphoea represents the commonest variant of LS, presenting as dermal sclerosis or calcifications from excessive collagen production and deposition.^1,6^ This is manifested as indurated areas of skin, typically on the trunk and limbs, and calcifications.^6^

Clinically, patients may present with skin discolouration, nodularity, pain and limited mobility depending on the location, as well as ulceration and superimposed infection.^1^ The condition is generally self-limiting with morbidity depending on the site of involvement (e.g., joints and tendons) and/or superimposed infection. The disease entity is commonly seen in middle-aged females.^1^ Plain radiography identifies the calcifications as first line imaging.^1^ Imaging with CT and MRI can also be utilised to assess bones and joints in cases of associated morbidity.^1^ MRI depicts the accompanying inflammatory changes in the fascia, muscles, tendons and bones.^5^ Confirmation with biopsy and histopathology, with calcium deposits identified in the dermis and subcutaneous tissues, is usually diagnostic.^1^

While breast calcifications, both benign and malignant, are not novel imaging findings, these more bizarre dystrophic calcifications hint at a systemic cause and should prompt further investigation.
